# Genetic Diversification of Tomato and Agricultural Soil Management Shaped the Rhizospheric Microbiome of Tomato (*Solanum lycopersicum*)

**DOI:** 10.3390/microorganisms13071550

**Published:** 2025-07-01

**Authors:** Máximo González, Juan Pablo Araya-Angel, Ashlie Muñoz, Adalid Alfaro-Flores, Massimiliano Cardinale, Alexandra Stoll

**Affiliations:** 1 Laboratorio Fisiología y Hologenómica Vegetal, Centro de Estudios Avanzados en Zonas Áridas, CEAZA, La Serena 1720256, Chile; 2 Departamento de Biología, Facultad de Ciencias, Universidad de La Serena, La Serena 1720256, Chile; jparaya@userena.cl (J.P.A.-A.); adalid.alfaro@userena.cl (A.A.-F.); 3 Laboratorio Microbiología Aplicada, Centro de Estudios Avanzados en Zonas Áridas, CEAZA, La Serena 1720256, Chile; 4 Department of Biological and Environmental Sciences and Technologies, University of Salento, SP6 Lecce-Monteroni, 73100 Lecce, Italy; massimiliano.cardinale@unisalento.it

**Keywords:** plant domestication, wild ancestors, landrace, rhizosphere microbiome, plant–microbe interactions

## Abstract

The domestication process not only reduced the allelic diversity of tomato genotypes but also affected the genetic traits associated to microbial recruitment, their composition, and their diversity in different compartments of the plant host. Additionally, this process included the transition from natural to agricultural soils, which differ in nutrient availability, physicochemical properties, and agricultural practices. Therefore, modern cultivars may fail to recruit microbial taxa beneficial to their wild relatives, potentially losing important ecological functions. In this study, we analyzed the phylogenetic relationship and the rhizosphere microbiota of four tomato genotypes, *Solanum chilense* (wild species), *S. lycopersicum* var. *cerasiforme* (Cherry tomato), and the *S. lycopersicum* landrace ‘Poncho Negro’ and the modern cultivar ‘Cal Ace’, grown in both natural and agricultural soils. Microbial communities were identified using 16S rRNA (bacteria) and ITS2 (fungi) amplicon sequencing, allowing cross-domain taxonomic characterization. While the soil type was the main driver of overall microbial diversity, the host genotype influenced the recruitment of specific microbial taxa, which exhibited different recruitment patterns according to the genetic diversification of *Solanum* genotypes and soil types. Additionally, co-occurrence network analysis identified two main clusters: first, taxa did not show any preferential associations to particular genotypes or soil types, while the second cluster revealed specific microbial patterns associated to fungal taxa in natural soil and bacterial taxa in agricultural soil. Finally, the functional analysis suggested the loss of specific functions through tomato domestication independently of soil type. These findings highlight the role of the plant genotype as a fine-tuning factor in microbiome assembly, with implications for breeding strategies aimed at restoring beneficial plant–microbe interactions.

## 1. Introduction

Plant phenotypes result from the intricate interplay of genotype, environment, and the associated microorganisms [1]. In a holobiont context, where the host and its associated microorganisms coexist, the microbial communities contribute to growth promotion, nutrient uptake, and biotic and abiotic stress tolerance, significantly enhancing the adaptation of the host to different ecosystems [2]. Recently, Sharp & Foster (2022) [3] challenged the holobiont’s traditional status as a single unit of natural selection. They argue that successful host–microbiome cooperation requires host control over symbionts, making genetic modifications a pivotal factor influencing traits related to microbiome recruitment. Therefore, plant genotype selection, through domestication and breeding, could not only systematically reduce the allele pool within chosen genotypes [4,5] but also affect the composition and diversity of the recruited microorganisms in the domesticated species and its cultivars [6,7], as a consequence of the modified phenotypes selected by the domesticator, such as larger fruits or bigger seeds [8].

In the rhizosphere, where roots and soil microorganisms constantly exchange energy and nutrients [9], microorganisms are recruited by root exudates, which vary according to different phenological stages and environmental conditions [2,10]. Due to the selection process, different genotypes have shown changes in root architecture and quantitative and qualitative changes in root exudates, among modern cultivars and relative species as well as genotypes within the same species [11,12]. This leads to different microorganisms being recruited among selected genotypes, landraces, and wild relatives [12,13]. Based on the contribution to their fitness, plants select those beneficial microorganisms at each step of the selection process, modifying the microbial assemblage as a result of the domestication from wild genotypes to modern cultivars [6].

In addition, the development of agricultural management involves the co-adaptation of plants from natural to fertilizer soils. Both types of soils differ in the pool of available nutrients and soil properties. Therefore, based on the requirement of the plant genotypes in each type of soil, the microbial recruitment of modern cultivars does not necessarily include those taxa that support plant fitness in their wild relatives, potentially losing important microbial taxa and their associated functions [7].

Tomato is one of the most important modern crops worldwide (http://faostat.fao.org). Its domestication involved the fully wild *Solanum pimpinellifolium*, the wild and semi-domesticated *S. lycopersicum* var. *cerasiforme* (Cherry tomato), and finally, the cultivated tomato *S. lycopersicum* [14]. Today, tomato cultivation relies on modern cultivars and landraces—varieties used by farmers and adapted to agroecological conditions—which serve as valuable sources of genetic traits for coping with harsh abiotic stresses such as salinity and drought [15,16]. This process involved not only the genetic diversification associated with cultivated tomato but also the transition from native to agricultural soils and management practices (e.g., fertilization and pesticides, among others), factors that have shaped the taxonomic and functional diversity of the microbiota in the tomato rhizosphere [17].

In this study, we hypothesized that the genetic diversification associated with tomato domestication and the transition from native to agricultural soils have reduced the taxonomic and functional diversity of the rhizospheric microbiota. Therefore, we compared the rhizosphere microbiota of tomato relatives, including the wild tomato *S. chilense*, a Cherry tomato (intermediate stage of domestication, *S. lycopersicum* L. var. *cerasiforme*), and two domesticated tomato types (*S. lycopersicum* L.): the landrace var. ‘Poncho Negro’ and the modern var. ‘Cal Ace’. Our results showed the taxonomic and functional changes involved in the tomato genetic diversification process, and demonstrated their relevance to plant fitness.

## 2. Materials and Methods

### 2.1. Plant Material

Four different genotypes were selected to assess the effect of genetic diversification on microbial recruitment. The wild relative *Solanum chilense*, adapted to the native zones of the northern of Chile [18]; the Cherry tomato *S. lycopersicum* L. var. *cerasiforme*; and two *S. lycopersicum* L. were included, specifically the landrace *S. lycopersicum* var. ‘Poncho Negro’ and the commercial cultivar *S. lycopersicum* var. ‘Cal Ace’ [19].

### 2.2. Phylogenetic Analysis

Double digest restriction-site-associated DNA (ddRAD) sequencing analysis was performed to determine the genetic relationships among all selected genotypes, including *S. pinnetum* as an outgroup. DNA samples were isolated using a DNeasy Plant Mini Kit (Qiagen, Hilden, Germany), following the manufacturer’s instructions. ddRAD analyses were performed by outsourcing at IGATechnology facilities (IGATechnology Inc., Udine, Italy). Libraries were prepared using the Peterson’s double digest restriction-site-associated DNA protocol, with minor modifications [20]. Raw Illumina reads were demultiplexed using the Stacks v2.53 software [21]. The FASTQ data were then aligned to the *S. lycopersicum* reference genome SL4.00 using BWA-MEM version 0.7.18-r1243-dirty [22]. Loci were identified and filtered from the aligned reads using the Stacks v2.53 software [21]. To visualize the genetic relationship among all samples, a neighbor-joining tree with 1000 bootstrap resamplings was constructed.

### 2.3. Experimental Design

The effect of the tomato genotype on microbial recruitment was evaluated on two contrasting soils: (1) natural (undisturbed) soil, collected in the south of the Atacama Desert (29°56′41.97″ S, 71°12′5933″ O, La Serena, Chile) (Supplementary Figure S1a), and (2) agricultural (cultivated) soil (29°54′10.06″ S, 71°12′35.87″ O, La Serena, Chile), corresponding to an intensively cultivated field (Supplementary Figure S1b). Ten seeds of each genotype were germinated on peat at 25 °C. After 30 days, 5 plants per genotype were transplanted to 500 mL pots filled with native or productive soil, respectively. The pots were distributed in a greenhouse using a completely randomized design and the microbial recruitment was evaluated after 30 days. At the end of the experiment, a composed soil sample for each soil type was collected to determine the concentrations (mg/kg) of the following: N (extracted with 2M KCL and quantified by titration), P (extracted with 0.5 M NaHCO_3_ at pH 8.5 and measured colorimetrically via the blue molybdenum complex protocol), K (extracted with 1 M ammonium acetate (NH_4_OAc) solution at pH 7.0 and quantified by atomic absorption spectrophotometry), organic matter (OM g/kg, determined by oxidation with 1 N potassium dichromate (K_2_Cr_2_O_7_) and colorimetric measurement of dichromate reduction), pH (determined in a 1:2.5 (*w*/*v*) soil–water suspension, using a calibrated glass-combined electrode), and electrical conductivity (EC, measured in a 1:5 (*w*/*v*) soil–water extract, using a conductivity meter). All analyses were performed by the Technological Centre of Soil and Cultivation [Centro Tecnológico de Suelo y Cultivo (CTSyC)], Talca, Chile (Supplementary Figure S2).

### 2.4. DNA Isolation, Amplicon Sequencing, and Bioinformatic Analysis

Three plants per genotype were randomly collected from both native and agricultural soils. Rhizospheric environmental DNA (eDNA) was extracted from the 24 samples using the PowerSoil DNA Isolation Kit (Qiagen), following the manufacturer’s instructions. eDNA integrity was assessed on a 1% agarose gel, and its purity and concentration were evaluated using a NanoDrop ND-1000 spectrophotometer (Thermo Scientific, Wilmington, DE, USA).

Bacterial and fungi communities were analyzed on the Illumina Miseq platform (San Diego, CA, USA), using the 16S rRNA gene (V3–V4 region: Forward-S-D-Bact-0341-b-S-17 5′-CCTACGGGNGGCWGCAG-3′ and Reverse-S-D-Bact-0785-a-A-21 5′-GACTACHVGGGTATCTAATCC-3′) [23] and the ITS2 region (Forward-ITS3 5′-GCATCGATGAAGAACGCAGC-3′ and Reverse-ITS4 5′-TCCTCCGCTTATTGATATGC-3′) [24], respectively. Amplicons were sequenced by Macrogen, Inc. (Seoul, Republic of Korea), resulting in paired-end reads of 300 pb. The raw reads are available on the NCBI database under the BioProject code PRJNA1224494 (Supplementary Table S1).

Bioinformatics analysis was performed using the QIIME2 software (version 2019.10) [25]. The raw reads were processed using the DADA2 pipeline to produce Amplicon Sequence Variants (ASVs) [26]. After chimera removal, 1,229,750 high-quality bacterial and 1,025,079 high-quality fungal reads were obtained (Supplementary Table S2), which were clustered into 4581 bacterial and 915 fungal ASVs, respectively. Taxonomic identification of bacteria and fungi were performed by aligning the ASVs to the SILVA v138 [27] and UNITE v.04.02.2020 [28] databases, respectively. Finally, ASVs with less than 20 sequences and those assigned to mitochondria, chloroplasts, and eukaryotes were removed.

### 2.5. Taxonomic, Alpha, and Beta Diversity

The ASVs were grouped at the taxonomic levels of phylum and genus. Phyla with a relative abundance greater than 1%, as well as the 20 most abundant genera across all samples, were selected for the construction of relative abundance bar plots.

On the other hand, a rarefaction curve was performed to identify the diversity captured for all samples (Supplementary Figure S3). For alpha and beta diversity analysis, the bacterial and fungal datasets were normalized by rarefaction [29], based on the minimum number of sequences identified in the samples (Supplementary Figure S4), which were 11,905 and 5.605 sequences for bacteria and fungi, respectively. The alpha diversity indices Shannon (H’), Faith’s phylogenetic diversity, Pielou’s evenness, and Observed ASVs were calculated. Between-samples similarity (beta diversity) was assessed using the UniFrac unweighted phylogenetic distance matrix and then represented in a Principal Coordinate Analysis (PCoA) plot [30].

### 2.6. Determination of Specific Taxa Patterns by Genotype and Functional Prediction

Differences in the ASV abundances due to genetic diversification or soil effect were identified using two approaches. First, a Self-Organization Map (SOM) analysis was performed. For this, the scaled abundance values within samples were used to cluster taxa for a multidimensional 3 × 2 hexagonal SOM throughout a 100 training iterations process using the Kohonen package on R [31]. To visualize the abundance patterns according to the genetic diversity or soil effect, a boxplot diagram was drawn for each node using the ggplot2 package on R [32]. A second approach, based on LefSe analysis using the microbiomeMaker package on R [33], was used to determine significant differential abundance patterns due to genetic diversity as well as the soil effect, natural or agricultural, per genotype. Among the compared conditions, only taxa with LDA scores > 2.0 were considered significant.

The functional diversity of bacterial communities was predicted using both FAPROTAX (Functional Annotation of Prokaryotic Taxa) v1.2.2 [34] and the Tax4Fun2 R package v1.1.5 [35]. In addition, the biological functions predicted by Tax4Fun2 were grouped according to level-1 pathway annotations and statistically compared using the software STAMP v2.1.3 [36].

### 2.7. Co-Occurrence Network Analysis

Co-occurrence network analysis was performed on the ASVs. The software CoNet [37], an add-on of Cytoscape 3.10 [38], was used to calculate the significant positive or negative connections based on the non-rarefied dataset of read counts. Only the 141 ASVs having more than 480 reads (20 × 24–the number of samples) and occurring at least in half of the samples (12) were considered for this network analysis. Pairwise scores were computed for Bray–Curtis distances, Kullback–Leibler dissimilarity, Pearson correlations, and Spearman correlations. For each of these measures, 100 permutations and 100 bootstrap-resampling scores were generated (with both renormalization and row-shuffling), and unstable edges were removed. The four *p*-values were merged based on Brown’s method (variances pooled by *p*-values’ combination of permutations and bootstraps). *p*-values were FDR-corrected with the Benjamini–Hochberg method and only highly significant correlations (*p*-value < 0.001), supported by at least three of the four similarity measures, were kept: this procedure compensates the method biases [37]. The network layout was visualized in Cytoscape, using the “edge-forced spring embedded” algorithm weighted by *p*-values [39], to obtain a network where interconnected nodes are placed closer to each other [40]. The network legend was created with the Cytoscape-App “Legend creator” (http://apps.cytoscape.org/apps/legendcreator, accessed on 24 June 2025), while Adobe Photoshop (Adobe Systems Inc., San Jose, CA, USA) was used to assemble the final figure.

### 2.8. Statistical Analysis

One-way ANOVA was used to detect significant differences (*p*-value < 0.05), and a Tukey post-hoc test was subsequently performed in the following analyses: relative abundance (phylum and genus), alpha diversity (at the ASV level), and abundance of functional groups detected by FAPROTAX. When ANOVA was significant, the Tukey post-hoc test was applied.

In the beta diversity analysis (unweighted UniFrac metrics), the significance of each factor (genotype and soil type) and their interaction was evaluated using the ADONIS method (QIIME2), considering a *p*-value < 0.05 and 999 permutations [41,42]. All statistical analyses were performed using R software (v4.0.2) [43].

## 3. Results

### 3.1. Phylogeny of the Selected Cultivars

ddRAD analysis identified 11,828 SNPs, whereas the phylogenetic analysis showed a consistent genetic relationship between the selected tomato genotypes. While *S. pinnatum* was identified as an outgroup, a near genetic relationship among both *S. lycopersicum* (‘Poncho Negro’ and ‘Cal Ace’) cultivars was confirmed, followed by Cherry tomato and *S. chilense*, respectively. Thus, the evaluated tomato genotypes showed a genetic relationship that was expected due to the genetic diversification process (Supplementary Figure S5).

### 3.2. Effect of Plant Genetic Diversity and Soil Type on the Tomato Rhizosphere-Associated Microbiota

#### 3.2.1. Taxonomical Diversity

**Figure 1:**
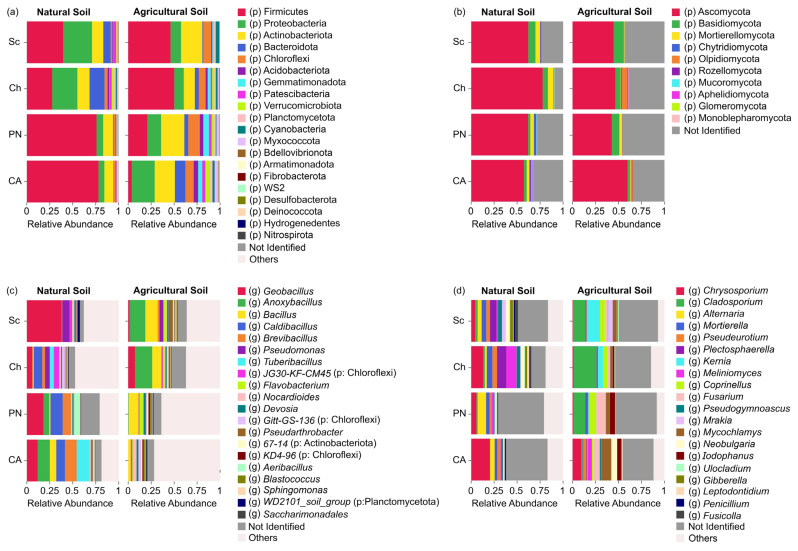
Comparison of relative abundances at the phylum level and of the 20 most abundant bacterial and fungal ASVs across different tomato genotypes grown in natural and agricultural soils. (**a**) Bacterial phyla; (**b**) fungal phyla; (**c**) 20 most abundant bacterial ASVs; (**d**) 20 most abundant fungal ASVs (Sc = *Solanum chilense*, Ch = *S. lycopersicum* var. *cerasiforme*, PN = *S. lycopersicum* var. ‘Poncho Negro’, CA = *S. lycopersicum* var. ‘Cal Ace’). The percentage of abundance for each taxon represents the mean of three biological replicates.

A total of 34 bacterial phyla with an abundance > 1% were identified, where Firmicutes, Proteobacteria, Actinobacteriota, Bacteroidota, Chloroflexi, Acidobacteriota, Gemmatimonadota, Patescibacteria, Verrucomicrobiota, Planctomycetota, Cyanobacteria, and Myxococcota represented 98.94% of the total sequences (Figure 1a). Although different patterns of relative abundance were observed among genotypes in each soil type, these were not significant (Supplementary Tables S3 and S4). However, differences were observed among genotypes due to the soil type effect. *S. chilense* and Cherry showed an enrichment in the phyla Proteobacteria and Bacteroidota in natural soil compared to agricultural soil, the soil type where *S. lycopersicum* cultivars showed a major abundance of these taxa (Figure 1a). Additionally, a similar pattern was observed in the phylum Firmicutes, which was highly abundant in ‘Poncho Negro’ and ‘Cal Ace’ in natural soil; meanwhile, the major abundance in *S. chilense* and Cherry was in agricultural soil (Figure 1a).

Ten fungal phyla were identified, namely Ascomycota, Basidiomycota, Mortierellomycota, Chytridiomycota, Olpidiomycota, Rozellomycota, Mucoromycota, Aphelidiomycota, Glomeromycota, and Monoblepharomycota (Figure 1b). In natural soil, the plant genetics only affected the abundance of Chytridiomycota and an unidentified phylum (Supplementary Tables S5 and S6), showing both taxa having a higher abundance in var. ‘Poncho Negro’ with respect to Cherry tomato (Figure 1b). In addition, specific recruitment due to the soil effect was observed. The phyla Olpidiomycota and Glomeromycota were recruited exclusively on agricultural and natural soils, respectively (Supplementary Tables S5 and S6).

On the other hand, the effect of soil type was observed in the bacterial microbiota at the genus level, with 660 genera and 92 uncultured taxa identified. *Tuberibacillus* was absent in the rhizosphere of all tomato genotypes grown in agricultural soil; however, the genus *Gitt-GS-136* (phylum: Chloroflexi) was absent in the rhizosphere of all genotypes evaluated in natural soil (Figure 1c). In addition, a selection of taxa and/or changes in their abundance among the different tomato genotypes occurred, which can be clearly observed comparing the taxa abundance of the more distant genotypes of genetic diversification. In ‘Cal Ace’–natural soil, high abundances of the genera *Anoxybacillus*, *Brevibacillus*, and *Caldibacillus* were observed, representing 13.19%, 11.83%, and 9.52%, respectively, while in the wild genotype *S. chilense*–natural soil, these genera were absent. However, in the ‘Cal Ace’–agricultural soil, the previously mentioned genera were absent, except for the genus *Brevibacillus*, with a low relative abundance of 0.003%. Contrasting results were observed in *S. chilense*–agricultural soil, where the genera *Anoxybacillus*, *Brevibacillus*, and *Caldibacillus* represented 17.18%, 2.09%, and 0.03% of the relative abundance, respectively (Figure 1c).

In fungi, 170 genera and 42 uncultured taxa were identified. The genotypes grown in agricultural soil showed a higher number of genera with respect to the natural soil, especially *Kernia*, *Coprinellus*, *Mycochlamys*, and *Iodophanus* (Figure 1d). In addition, the genus *Gibberella* was present only in the rhizosphere of *S. chilense* and Cherry, but not in *S. lycoperscum* cultivars.

#### 3.2.2. Alpha and Beta Diversity

**Figure 2:**
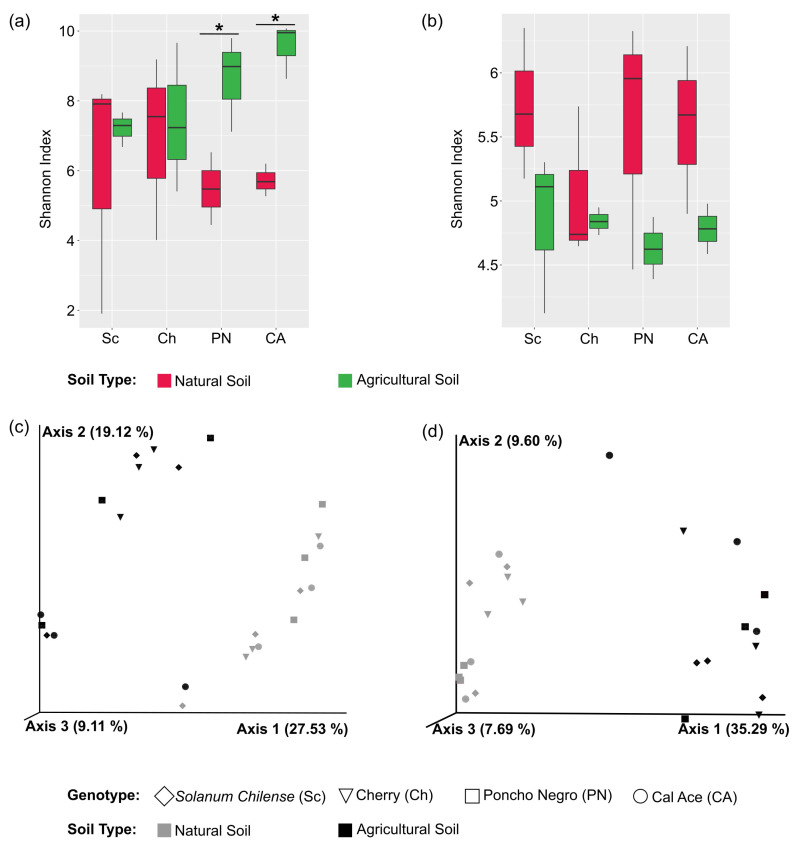
Alpha and beta diversity analyses based on the four evaluated genotypes and both soil types (agricultural and natural). Panels (**a**,**b**) show Shannon diversity indices for bacteria and fungi, respectively. Asterisks (*) indicate statistically significant differences detected by ANOVA at *p*-value < 0.05. Significant differences were only observed when comparing each genotype across the two soil types. Panels (**c**,**d**) present the Principal Coordinate Analysis (PCoA) based on unweighted UniFrac distances for bacterial and fungal communities, respectively.

Independently from soil type, tomato genotype diversification was associated with non-significant differences in alpha diversity (Supplementary Tables S7 and S8 for bacteria and fungi, respectively). However, in agricultural soil, bacteria tended to show an increase in all diversity indices, from *S. chilense* to ‘Cal Ace’ (Figure 2a; Supplementary Figure S6), a pattern that was not observed in fungi (Figure 2b).

Alpha diversity indices only showed significant differences when each tomato genotype was compared between both soils. Shannon’s diversity index (H’) showed that the bacterial microbiota present in *S. lycopersicum* cultivars ‘Cal Ace’ and ‘Poncho Negro’ have higher diversity in the agricultural soil type compared to natural soil (Supplementary Figure S6; Supplementary Tables S9 and S10). In contrast, in fungi, it was observed that the highest alpha diversity occurs in natural soil with ‘Cal Ace’ and ‘Poncho Negro’ (Supplementary Figure S6; Supplementary Tables S11 and S12).

On the other hand, beta diversity analysis showed that bacteria and fungi microbial communities had a significant grouping of samples according to soil type (*p*-value < 0.001) (Supplementary Tables S13 and S14, respectively), explaining the 27.53% and 35,29% of PC1 in the PCoA performed for each kind of microorganism (Figure 2c,d, for bacteria and fungi, respectively). When the effect of tomato genotype on bacterial communities was analyzed, no significant differences were identified regarding the factor ‘Genotype’ and the ‘Soil type*Genotype’ interaction (Supplementary Tables S15 and S16).

#### 3.2.3. Effect of Tomato Genotype and Soil Type on Microbial Abundance

**Figure 3:**
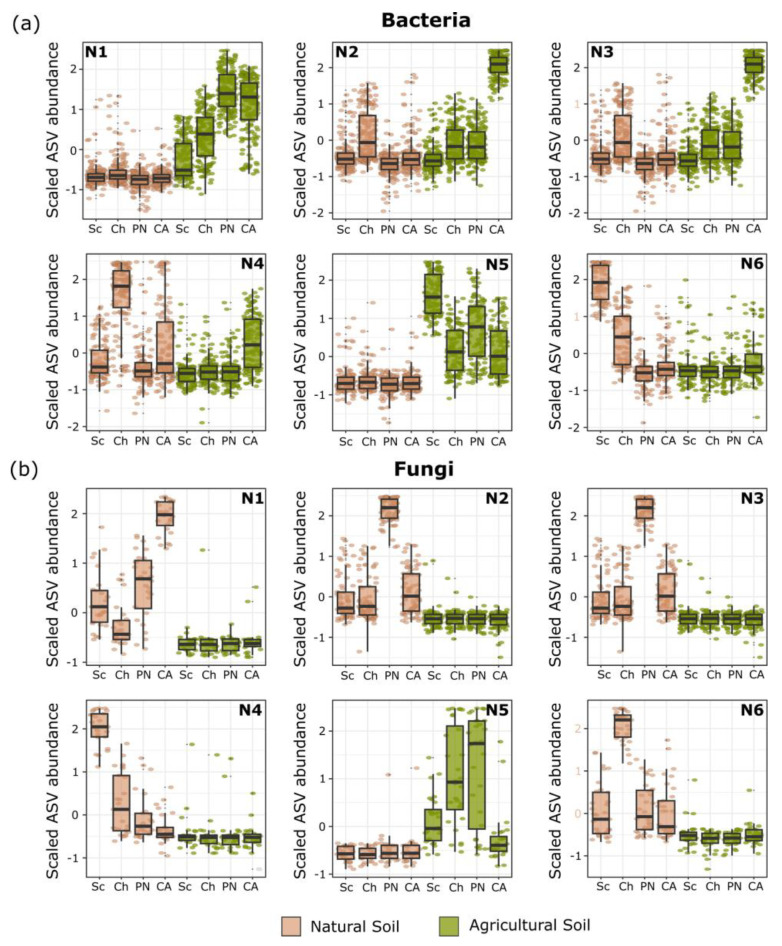
Taxa abundance patterns identified through self-organizing map (SOM) analysis. The SOM analysis generated six distinct nodes, each representing a unique pattern of taxa abundance associated with specific genotype and soil type combinations. Panels (**a**,**b**) correspond to bacterial and fungal communities, respectively. Tomato genotypes: Sc = *Solanum chilense*, Ch = *S. lycopersicum* L. var. *cerasiforme*, PN = *S. lycopersicum* L. var. ‘Poncho Negro’, CA = *S. lycopersicum* L. var. ‘Cal Ace’.

To determine the effect of the tomato genotype and the soil type on the abundance of the recruited microorganisms, an SOM analysis was performed (Figure 3). In bacteria, Node 6 (N6) grouped those phyla that are reduced from *S. chilense* to ‘Cal Ace’ in natural soil. In addition, in this node, it was possible to identify the unique phylum Abditibacteriota. On the other hand, Node 1 (N1) showed recruitment patterns in agricultural soil according to the genetic diversification, highlighting the specific phyla Nitrospirota and SAR324_clade (Marine_group_B). Finally, in the same soil, Node 5 (N5) suggests a number of taxa are diminished according to the diversification, being high in *S. chilense* but reduced in Cherry, ‘Poncho Negro’, and ‘Cal Ace’, being found exclusively in the phylum Deinococcota (Supplementary Table S17).

Among fungi, the phyla Ascomycota and Basidiomycota were present in all nodes. Only in Node 5 was it possible to identify an abundance pattern associated with tomato diversification in agricultural soil, with the phylum Olpidiomycota found exclusively in this node. Meanwhile, nodes N1 and N4 exhibited contrasting patterns—with ASV abundance increasing in N1 and decreasing in N4—according to the domestication pattern observed in natural soil. Additionally, the phylum Aphelidiomycota was exclusive to N1 (Supplementary Table S18).

**Figure 4:**
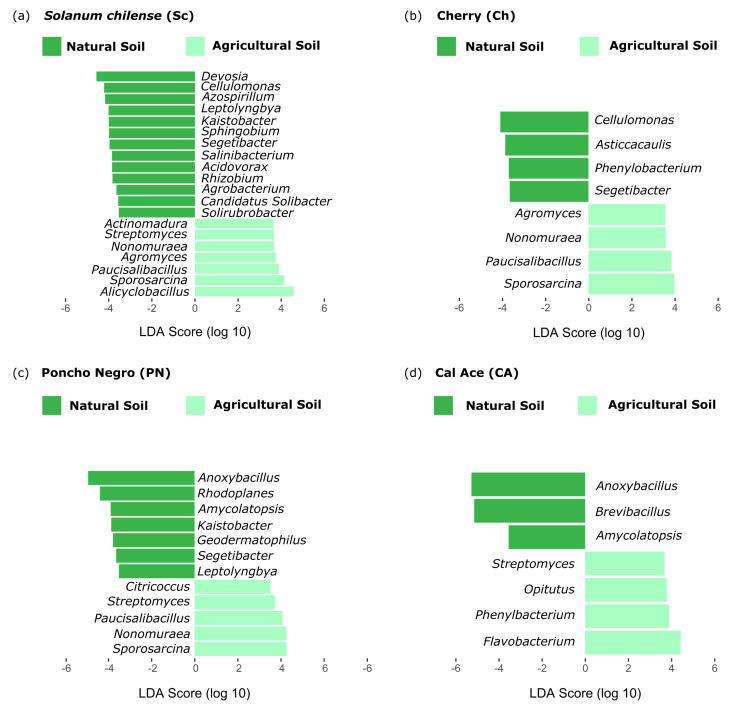
Linear Discriminant Analysis Effect Size (LefSe) identifying preferential bacterial taxa at the genus level in the rhizosphere of four tomato genotypes grown in both natural and agricultural soils. Only taxa with an LDA score > 2.0 are shown.

Similar results were observed on LefSe analysis, where the significantly abundant taxa diminished according to the genetic diversification process (Figure 4). The bacterial genus *Cellulomonas* was significantly abundant in natural soil with respect to agricultural soil in *S. chilense* and Cherry. Also, our results in natural soil showed the presence of the genus *Segetibacter* in *S. chilense*, Cherry, and ‘Poncho Negro’, but not in ‘Cal Ace’. Finally, *Anoxibacillus* was observed just in the *S. lycopersicum* cultivars. The analysis on agricultural soil identified that the four most abundant taxa in Cherry are present in *S. chilense*, while three of them (*Nonomuraea*, *Paucisalibacillus*, and *Sporosarcina*) can also be found in ‘Poncho Negro’, but not in ‘Cal Ace’ (Figure 4).

**Figure 5:**
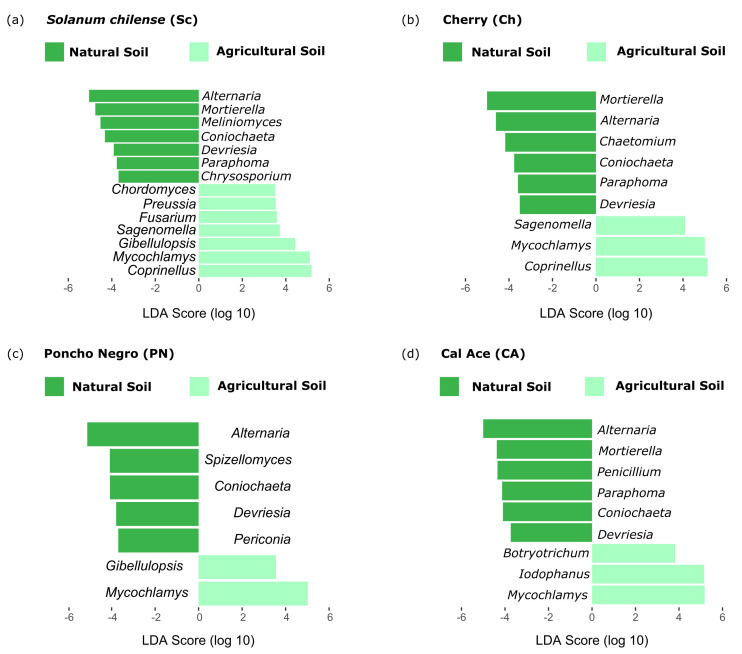
Linear Discriminant Analysis Effect Size (LefSe) identifying preferential fungi taxa at the genus level in the rhizosphere of four tomato genotypes grown in natural and agricultural soils. Only taxa with an LDA score > 2.0 are shown.

On the other hand, LefSe analysis in fungi consistently revealed a high abundance of the genera *Alternaria*, *Coniochaeta*, and *Devriesia* across all genotypes in natural soil. Additionally, specific fungal taxa were associated with distinct genotypes: *Chrysosporium* with *S. chilense*, *Chaetomium* with Cherry, *Spizellomyces* with ‘Poncho Negro’, and *Penicillium* with ‘Cal Ace’. In agricultural soil, only the fungal taxon *Mycochlamys* was consistently present across all samples. Meanwhile, the genera *Sagenomella* and *Coprinellus* were exclusively found in *S. chilense* and Cherry, respectively, *Gibellulopsis* was unique to ‘Poncho Negro’, and *Botryotrichum* and *Iodophanus* were identified solely in ‘Cal Ace’ (Figure 5).

#### 3.2.4. Co-Occurrence Patterns of Bacterial and Fungal Taxa

**Figure 6:**
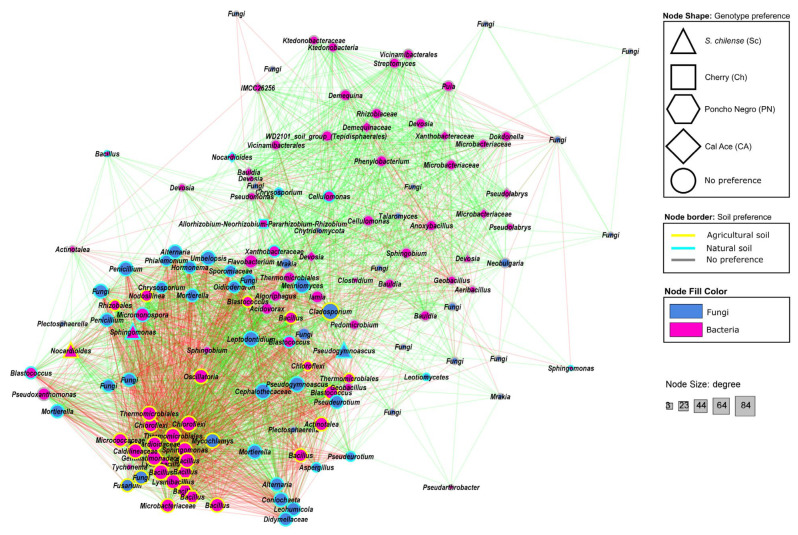
Co-occurrence network of bacterial and fungal taxa identified in the rhizosphere of four tomato genotypes cultivated in natural and agricultural soils. The network displays statistically significant correlations among microbial taxa, emphasizing potential interactions between bacterial and fungal communities across different genotype–soil type contexts. Nodes represent individual taxa, while edges indicate significant positive (green) or negative (red) correlations.

To identify co-occurrence patterns at the genus level, a combined bacteria and fungi network analysis was performed (Supplementary Tables S19 and S20; Figure 6). A total of 85 bacterial and 56 fungal ASVs showed significant correlations (*p*-value < 0.001), as determined by at least three out of four of the statistical methods tested. A total of 3073 interactions were identified. The taxa with the highest degree of interaction were the fungal genus *Cladosporium* (phylum: Ascomycota), with 84 interactions (56 negative and 28 positive), followed by *JG30-KF-CM45* (phylum: Chloroflexi), with 73 interactions (36 negative and 37 positive), and *Bacillus* (phylum: Firmicutes) with 72 interactions (34 negative and 38 positive).

The co-occurrence network displayed two main clusters (Figure 6). In both clusters, most taxa showed no preference for any tomato cultivar. Nonetheless, some exceptions were the following: *S. chilense—Nocardioides* (agricultural soil), *Sphingomonas*, and *Pseudogymnoascus* (both natural soil); Cherry—*Allorhizobium*, *Neorhizobium*, *Pararhizobium*, and *Rhizobium* (natural soil); ‘Poncho Negro’—*Bacillus* (agricultural soil); and ‘Cal Ace’—*Devosia* and *Pseudolabrys* (no soil preference) and *Nocardioides* (natural soil). In the first cluster, the positive correlation among bacterial ASVs with no clear preference for soil type or tomato genotype prevailed. The second cluster comprised bacterial and fungal taxa which demonstrated an overlapping pattern of positive and negative correlations. As mentioned before, most of the ASVs did not prefer a specific tomato genotype. However, this cluster revealed clear soil preferences of the involved microbial groups: distributed as a fan, the fungal ASVs associated to natural soil were positively correlated among each other, but negatively correlated with the bacterial ASVs; meanwhile, the bacterial ASVs were associated to agricultural soil and formed a solid subcluster, again with positive correlations among them and negative correlations to the fungal ASVs from natural soil.

#### 3.2.5. Functional Analysis of Bacterial Communities in the Tomato Rhizosphere

Based on FAPROTAX analysis, it was possible to identify 201 genera related to 39 functional groups. Of them, 12 functional groups (nitrate denitrification, nitrite denitrification, nitrous oxide denitrification, denitrification, and nitrite respiration, among others) showed significant differences among all samples (*p*-value < 0.05; Supplementary Figure S7). Through tomato domestication, biological processes related to the nitrogen cycle (denitrification of nitrate, nitrite, nitrous oxide, and nitrite respiration) were lost in both soil types. At the same time, the loss of functional groups related to aerobic chemoheterotrophy was also observed in the natural soil (Supplementary Figure S7).

To determine the genotype–soil effect through the diversification process, microbial functions associated with the more distant genotypes ‘Cal Ace’ and *S. chilense* were analyzed. A PCA analysis, based on Tax4Fun2 results, revealed in ‘Cal Ace’ differences in functional traits of the bacterial communities recruited on natural and agricultural soils; meanwhile, differences due to the soil type were not clearly observed in *S. chilense*. In addition, the recruited microbial functions were different between both genotypes independently of the soil type (Supplementary Figures S8 and S9a).

On the other hand, comparing the functional recruitment categories of ‘Cal Ace’ in natural versus agricultural soil, it was possible to identify that natural soil increased different carbohydrate processes (glycolysis/gluconeogenesis and fructose and mannose metabolism), as well as processes associated to the biosynthesis of amino acids, including lysine biosynthesis, alanine, aspartate, and glutamate metabolism, and cysteine and methionine metabolism. In agricultural soil, this genotype increased the categories of valine, leucine, and isoleucine biosynthesis, but also the bacterial secretion system, vancomycin resistance, and biofilm formation (*E. coli* and *Pseudomonas*) (*p*-value < 0.05) (Supplementary Figure S9b). In *S. chilense*, the communities in natural soil increased the categories of valine, leucine, and isoleucine biosynthesis, bacterial secretion system, oxidative phosphorylation, and flagellar assembly; meanwhile, agricultural soil highlighted inositol phosphate metabolism, nucleotide excision repair, and naphthalene degradation (Supplementary Figure S8c). Our results highlight that ‘Cal Ace’–agricultural soil as well as *S. chilense*–natural soil showed an increase of the categories alanine, aspartate, and glutamate metabolism and oxidative phosphorylation (Supplementary Figure S9b,c), suggesting that both functional categories could be a specificity between the genotype and their most adapted soil type.

## 4. Discussion

In plants, research on genetic diversity and domestication typically focuses on the acquisition of agriculturally important traits and the genetic erosion associated with this process [5]. It is now well understood that some traits are modified during speciation, selection, and domestication processes, involving changes in the microorganisms that plant hosts can recruit [6]. This shift can be attributed to changes in the metabolite profiles exuded by plant roots, which have been observed to vary more with diversification than with soil type in tetraploid wheat (*Triticum turgidum*) [12]. Whereas Huang et al. [44] reported that the domestication and genetic improvement process gradually increased the rhizobacterial diversity from teosinte, maize landraces, and modern maize, Pérez-Jaramillo et al. [44,45,46] determined that there are no significant differences in the alpha diversity between wild and modern accessions of *Phaseolus vulgaris*. At the same time, studies on wheat, maize, legumes, and barley have suggested that the domestication process reduces the taxonomic and functional diversity of the microbial communities recruited in the rhizosphere of cultivated plants [47,48,49]. Therefore, in microorganism and plant genetic diversity studies, usually three possible scenarios are concluded: the recruitment microbiome diminishes, increases, or is stable among wild relatives and cultivated species. However, the authors also suggest that the soil type effect must be included to determine if the observed results in their work can be refined [6,44].

This study aimed to determine the microbial recruitment patterns associated with the diversification process within the *Solanum* genus, from wild relatives to cultivated species, considering the effects of genotype and soil type. Our results revealed a trend toward increased alpha diversity in accordance with the co-adapted soil type (natural or agricultural soils, respectively). Specifically, the *S. lycopersicum* cultivars ‘Poncho Negro’ and ‘Cal Ace’ exhibited a significant increase in alpha diversity when grown in agricultural soil compared to natural soil. In contrast, no significant differences in fungal alpha diversity were observed across the analyzed genotypes. Beta diversity analysis indicated that, for both bacteria and fungi, soil type was the only statistically significant factor (Supplementary Table S13). In wheat, Simonin et al. [50] determined that the culturing of soil and agricultural practices are the main drivers of the microbiome, explaining 57% and 10% of the variance, respectively, while the genotypes had limited effects on microbiome diversity and structure. Similar results were observed by Pérez-Jaramillo et al. [45], who identified that the most relevant factor was soil type, explaining 71.3% of the total variability. In soybeans, Liu et al. [51] found that both the soil type and genotype contribute to microbiome assembly. While the soil type plays a dominant role in shaping the rhizosphere microbiome, the host genotype fine-tunes this recruitment process. These results highlight the importance of including the soil type as a factor in the experimental design in plant–microorganism studies, mainly when these studies involve species that have evolved associated with agricultural management.

On the other hand, the bacterial phyla Proteobacteria and Bacteroidota were consistently more abundant in the rhizosphere of genotypes grown in their adapted soil type, while the phylum Firmicutes was more prevalent in non-adapted soils. These three phyla are known to harbor bacteria with plant growth-promoting activities, such as *Pseudomonas* (e.g., siderophore production, root hair promotion, and tolerance to biotic and abiotic stresses), *Flavobacterium* (e.g., phosphate solubilization), and *Bacillus* (e.g., biotic stress response), respectively [17]. In addition, the bacterial genus *Tuberibacillus* (phylum: Firmicutes) was detected in all genotypes only in natural soil, while *Gitt-GS-136* (phylum: Chloroflexi) was identified exclusively in agricultural soil. These results suggest that plants may recruit microbial taxa according to their affinity for a given soil type, whereby certain PGPRs may be selected depending on the association, or lack thereof, between genotype and specific soil conditions. At the same time, in fungi, the phyla Olpidiomycota and Glomeromycota showed exclusive recruitment on agricultural and natural soils, respectively. Specifically, the phylum Glomeromycota includes the arbuscular mycorrhizal fungi, which are associated to different benefits in plants growth promotion, such as enhanced nutrient uptake and increased tolerance to biotic and abiotic stress, among others [52]. Therefore, our results suggest that, in soils that are not usually under agricultural practices, plants need to recruit microorganisms that support their fitness.

The microbial recruitment, based on domestication patterns, can be analyzed through gained or lost taxa, where the plant genotype plays a tuning role in microbial assembly [51], recruiting microbial taxa based on specific genetic traits, such as Quantitative Trait Loci (QTLs), as described for tomato by Oyserman et al. [53]. In order to further explore specific recruitment based on abundance patterns, SOM and LefSe analyses were performed. The first analysis was used to cluster each microorganism according to its abundance profile, identifying recruitment patterns associated with genotype and soil type. In this analysis, Node 1 (N1) revealed phyla such as Nitrospirota with a gradually increasing abundance according to adaptation to agricultural soil, which could be associated with nitrification processes that enhance nitrogen availability for plants and other microorganisms [54]. However, despite the gradually increasing abundance of Nitrospirota, the functional analysis determined that, through tomato domestication, biological processes related to the nitrogen cycle are lost in both soil types, suggesting that the presence of this taxon is not sufficient to alter this functional pattern.

At the same time, LefSe analysis allowed the identification of microorganisms whose abundance is associated with a genotype–soil type relationship. Microorganisms such as *Paucisalibacillus* and *Sporosarcina*, which have been described as PGPRs in previous studies [55,56], were present in *S. chilense*, Cherry, and ‘Poncho Negro’, but not in ‘Cal Ace’ under agricultural soil conditions. In this soil type, it was also possible to identify that three of the evaluated genotypes recruited microorganisms from the genus *Streptomyces*, while only ‘Cal Ace’ recruited *Flavobacterium*. Both genera are described as PGPRs: *Streptomyces* is likely recruited in association with the *Solanum* genus, whereas *Flavobacterium* (phylum: Bacteroidota) may be specifically recruited to maintain plant fitness in the co-adapted to agricultural soil type. This genus contributes to plant fitness through the induction of tolerance to biotic and abiotic stresses (e.g., induction of volatile organic compounds (VOCs)), as well as growth promotion via the synthesis of hormones such as indole-3-acetic acid and abscisic acid [57].

The co-occurrence network of the microbial communities associated to *S. lycopersicum* (tomato) genotypes grown in different soil types provided valuable insights into their ecological dynamics. The network displayed two major clusters. The first cluster predominantly contained bacterial ASVs positively correlated among each other with no apparent preference for the soil type or tomato genotype. In the literature, such resilient bacterial ASVs are considered generalists [58,59], which play an important role for maintaining microbial community stability in the rhizosphere [58], and provide redundancy in key functions such as nutrient cycling and disease suppression [60].

The second cluster revealed an interesting pattern of two overlapping subgroups: a group of fungal ASVs exclusively co-occurred in the natural soil, and a group of bacterial ASVs exclusively correlated in the agricultural soil. Both microbial groups are entirely negatively correlated with each other. Despite some publications comparing different soil management strategies (e.g., organic vs. conventional management) [61,62,63] or crop domestication in one type of soil [58,64], no comparable studies to our approach could be found. Regarding the group of bacterial ASVs in the agricultural soil, our results align with Chen et al. [65], who found that regular tillage enhanced bacterial generalists and impaired fungi. Nonetheless, contrary effects have also been reported for other crops [61,66]. The observed profound changes in keystone taxa from the natural soil to the agricultural soil also alters the functionality of the microbial community [61,63,67]. Additionally, agricultural management affects physicochemical soil properties such as pH, nutrient availability, moisture, and organic matter content, which also alter the composition and functionality of the soil/rhizosphere microbial community [68,69].

Finally, our results highlight that microbial communities are altered through domestication, with gains, losses, or the conservation of specific taxa resulting from this process. In tomato, Smulders et al. [70] studied the influence of genotype (plant ancestors, landraces, and domesticated cultivars) and reported that eight bacterial metabolic pathway categories changed along the domestication gradient, emphasizing the role of host genotype in shaping the functional potential of soil bacterial communities. Smulders et al. [70] reported that branched-chain amino acid metabolism is reduced because of domestication. However, we found that this response is highly dependent on soil type. Both ‘Cal Ace’ and *S. chilense* increased branched-chain amino acid metabolism in their most pre-adapted soil type (agricultural and natural soils, respectively). Additionally, the same authors found that nitrogen, sulfur, cofactor/vitamin, and purine metabolism decreased in modern cultivars compared to wild varieties. This result is consistent with our study, where tomato domestication led to the loss of nitrogen cycle functions (e.g., denitrification and nitrite respiration) in both soil types, likely explained by the co-adaptation of modern cultivars to agricultural practices such as chemical fertilization. On the other hand, studies in wheat suggest an increase in nitrogen-related functions with domestication [71]; at the same time, other studies have described that wild wheat cultivars can harness rhizosphere microorganisms involved in nitrogen transformations (i.e., nitrification and denitrification), whereas domesticated cultivars are associated with functions like inorganic nitrogen fixation and organic nitrogen ammonification [7]. Therefore, metagenomic (shotgun) approaches are required to more precisely identify which nitrogen cycle steps are lost or retained during domestication on the evaluated genotypes.

## 5. Conclusions

In conclusion, this work reinforces the idea that, while diversity indices show that the main differences in microbial recruitment can be explained by soil type, the recruitment of individual taxa can be attributed to host genetic patterns, suggesting a fine-tuning role of this factor in the microbial assembly. Therefore, new studies considering environmental factors (different soil types) and the inclusion of more genotypes will allow the identification of specific genetic traits associated with microbial recruitment, as well as the contribution of these microorganisms to the host traits. Additionally, metagenomic analyses may further reveal microbial functions recruited in wild cultivars versus landraces and commercial cultivars, helping to identify which functions could be specific to the co-adapted soil of each genotype.

## Data Availability

Raw reads have been deposited in the NCBI Sequence Read Archive (SRA) under BioProject ID PRJNA1224494.

## Supplementary Materials

The Supplementary Figures can be downloaded at https://www.mdpi.com/article/10.3390/microorganisms13071550/s1. Figure S1. Soil types used in this research. (a) Natural soil, similar to the native soil of wild ancestral tomato plants such as *S. chilense* (soil collected from Cerro Grande, La Serena, Chile); (b) agricultural soil, the same as natural soil but subject to intensive agricultural activity (soil collected from Coquimbito, Coquimbo, Chile). Satellite images were captured from Google Earth Pro, 2020 Maxar Technologies^®^. Figure S2. Physicochemical soil parameters of the natural and agricultural soils used in this study. Figure S3. Sequencing depth and diversity captured in all samples. (a) Bacterial samples; (b) fungal samples. Diversity was represented by the Shannon index. Figure S4. Rarefaction curves for all bacterial and fungal samples. (a) Bacterial samples rarefied to a maximum of 11905 ASV sequences per sample; (b) Fungal samples rarefied to a maximum of 5605 ASV sequences per sample. Diversity was represented by the Shannon index. Supplementary Figure S5. Genetic relationships between the tomato genotypes used in this study. Distances were assessed by SNPs identified using a Double digest restriction-site associated DNA (ddRAD) sequencing analysis, and *Solanum pinnatum* was included as outgroup. Figure S6. Alpha diversity indices. (a) Observed ASVs (bacteria); (b) Observed ASVs (fungi); (c) Pielou’s evenness (bacteria); (d) Pielou’s evenness (fungi); (e) Faith’s phylogenetic diversity (PD) (bacteria); (f) Faith’s phylogenetic diversity (PD) (fungi). Bars represent the average of three replicates, and error bars represent the standard deviation. * Significant differences detected by ANOVA (*p*-value < 0.05). Figure S7. Functional prediction according to FAPROTAX analysis. Relative abundance heatmap, showing functional groups that differ significantly between samples (Fold-Change 2, *p*-value < 0.05). Figure S8. Comparison of pathway prediction profiles between bacterial microbiota of selected genotypes visualized through PCA. (a) ‘Cal Ace’ in natural and agricultural Soils; (b), *S. chilense* in natural and agricultural soils; (c) ‘Cal Ace’ and *S. chilense* in natural soil; (d) ‘Cal Ace’ and *S. chilense* in agricultural soil. Figure S9. Comparison of pathway prediction profiles between bacterial communities of selected genotypes. (a) PCA of pathway predictions between ‘Cal Ace’ and *S. chilense* in both soil types. Mean proportion (%) and differences in the mean proportions (%) of the predicted pathway of bacterial communities of the (b) ‘Cal Ace’ and (c) *S. chilense* genotypes in natural and agricultural soils. On the other hand, Supplementary Tables S1–S20 associated to the raw data, DADA2 results, and differential statistical analysis.
