# Supplementary material for: Genetic Diversification of Tomato and Agricultural Soil Management Shaped the Rhizospheric Microbiome of Tomato (Solanum lycopersicum)
Source: Microorganisms. 2025 Jul 1;13(7):1550. doi: 10.3390/microorganisms13071550 (PMC12298371; doi:10.3390/microorganisms13071550)
Supplement: Supplementary file 1 [file microorganisms-13-01550-s001.zip › Supplementary figures_MG.pdf]

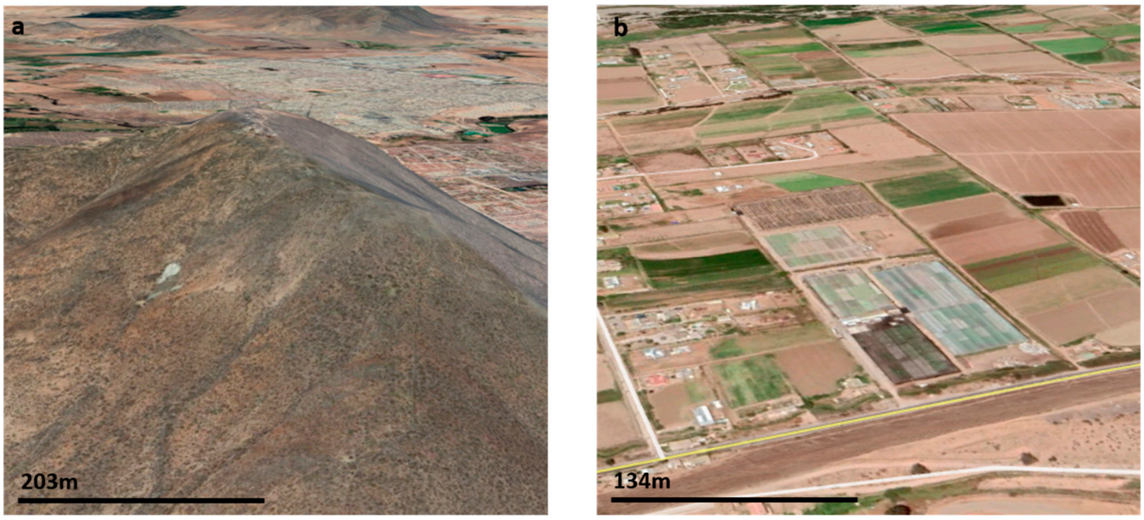

Supplementary Figure S1. Soil types used in this research. (a) Natural Soil, similar to the native soil of wild ancestral tomato plants such as *S. chilense* (soil collected from Cerro Grande, La Serena, Chile); (b) Agricultural Soil, the same as natural soil but subject to intensive agricultural activity (soil collected from Coquimbito, Coquimbo, Chile). Satellite images were captured from Google Earth Pro, 2020 Maxar Technologies®.

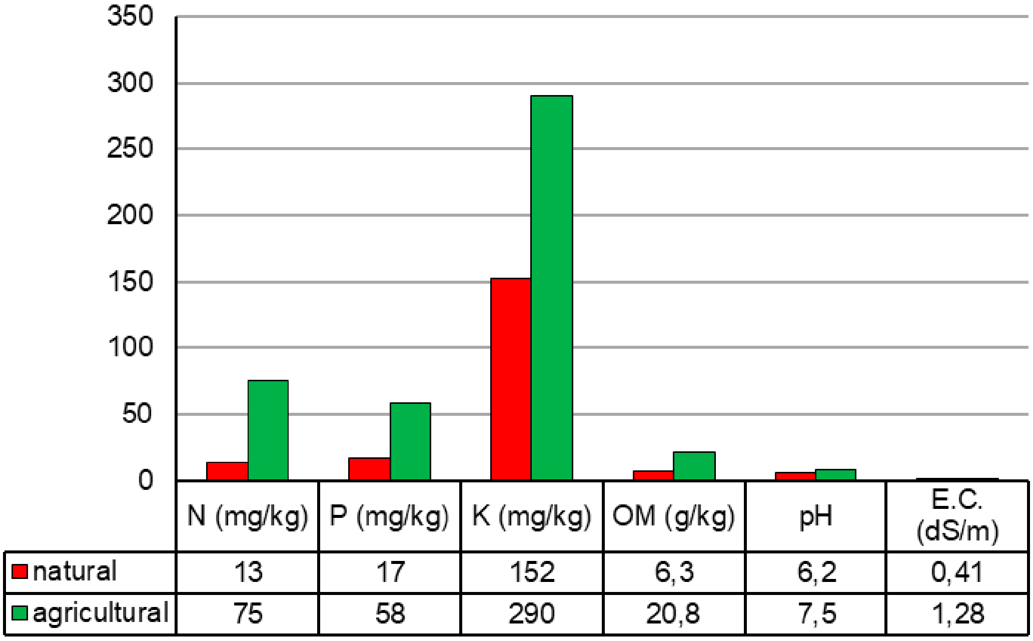

	N (mg/kg)	P (mg/kg)	K (mg/kg)	OM (g/kg)	pH	E.C. (dS/m)
natural	13	17	152	6,3	6,2	0,41
agricultural	75	58	290	20,8	7,5	1,28

Supplementary Figure S2. Physicochemical soil parameter of the natural and agricultural soil used in this study.

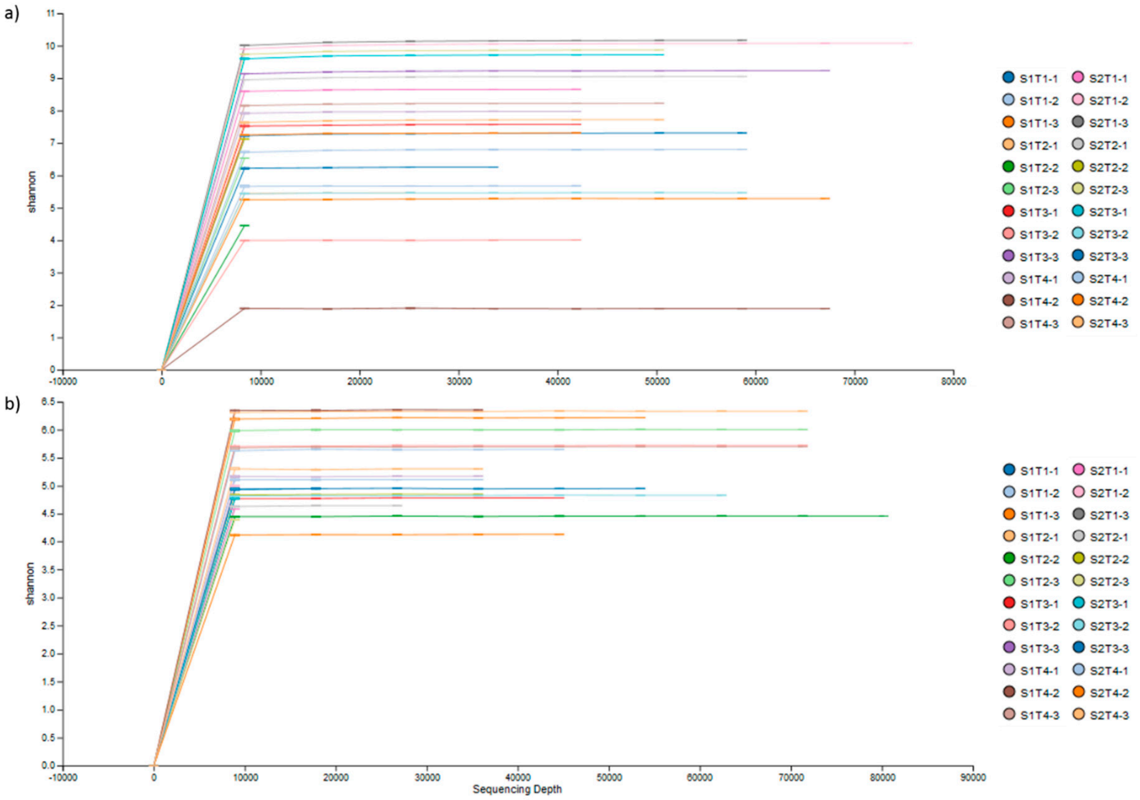

Supplementary Figure S3. Sequencing depth and diversity captured in all samples. (a) bacterial samples; (b) fungal samples. Diversity was represented by the Shannon index.

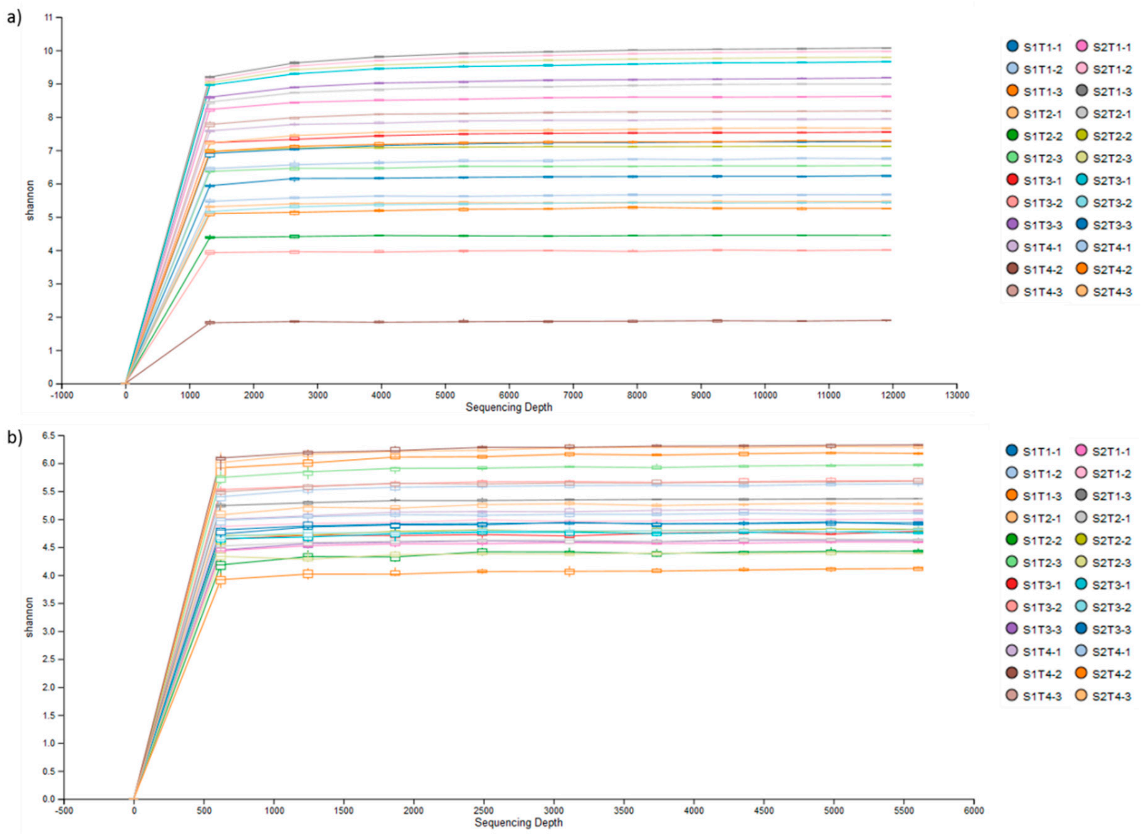

Supplementary Figure S4. Rarefaction curves for all bacterial and fungal samples. (**a**) Bacterial samples rarefied to a maximum of 11905 sequences per sample; (b) Fungal samples rarefied to a maximum of 5605 sequences per sample. Diversity was represented by the Shannon index.

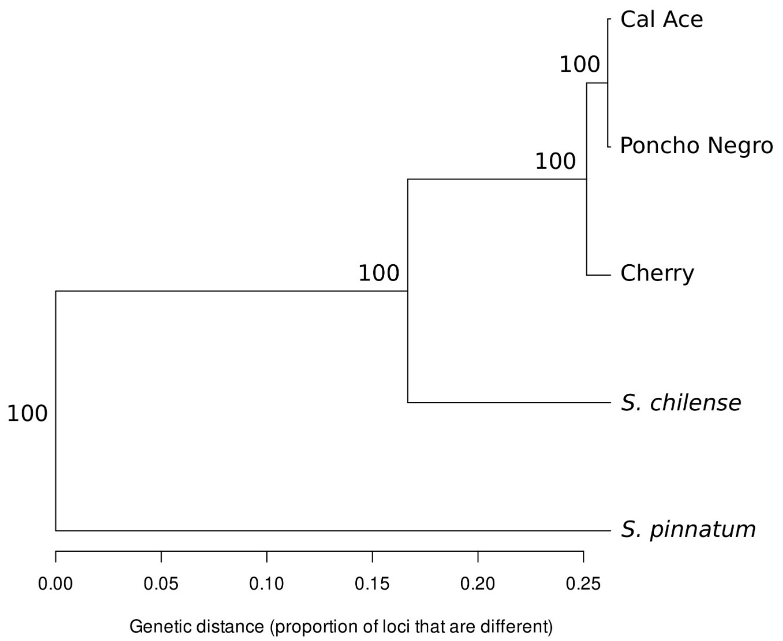

Supplementary Figure S5. Genetic relationships between the tomato genotypes used in this study. Distances were assessed by SNPs identified using a Double digest restriction-site associated DNA (ddRAD) sequencing analysis, and *Solanum pinnatum* was included as outgroup.

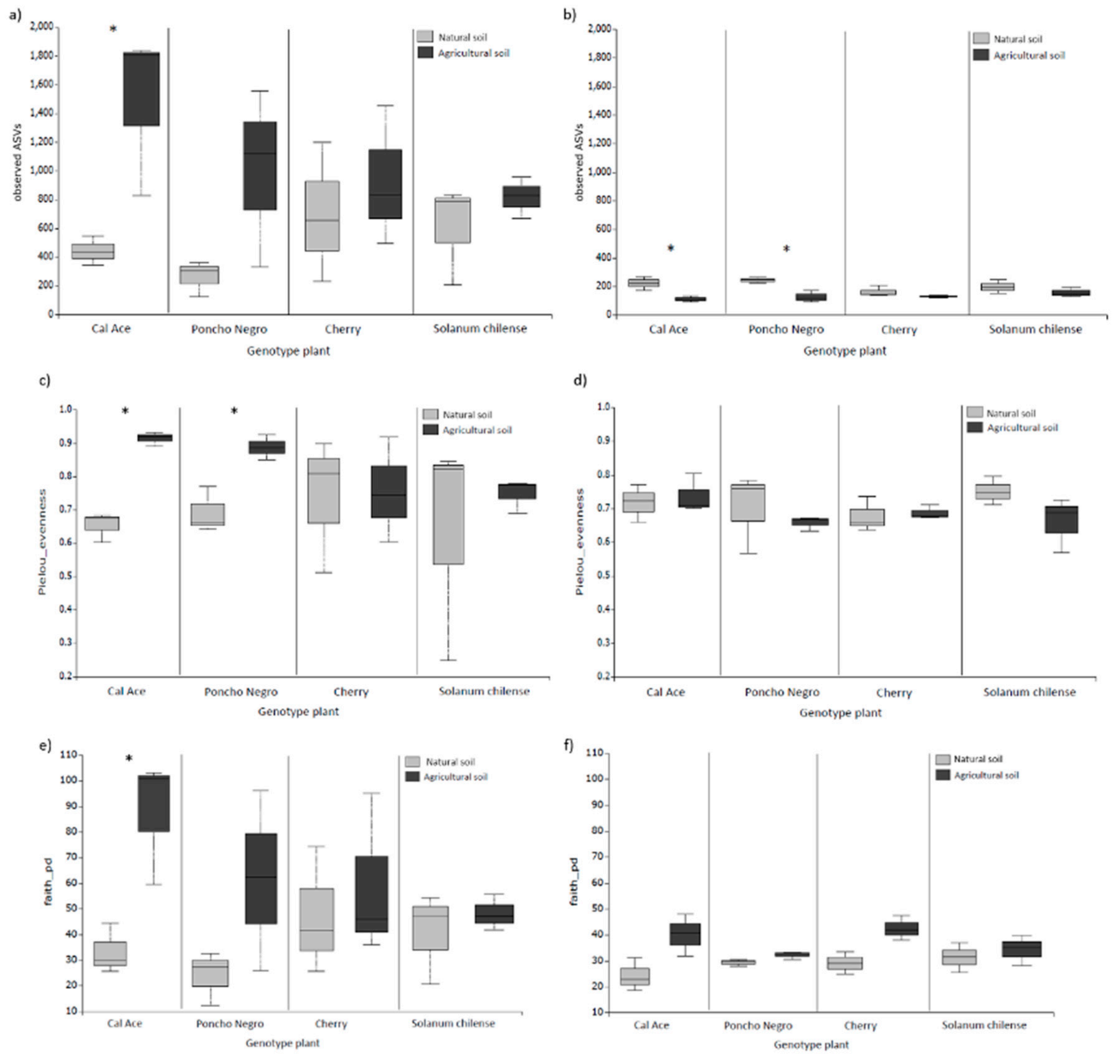

Supplementary Figure S6. Alpha diversity indices. (a) Observed ASVs (bacteria); (b) Observed ASVs (fungi); (c) Pielou's evenness (bacteria); (d) Pielou's evenness (fungi); (e) Faith's phylogenetic diversity (PD) bacteria); (f) Faith's phylogenetic diversity (PD) (fungi). Bars represent the average of three replicates, and error bars represent standard deviation. *Significant differences detected by ANOVA (*p-value* < 0.05).

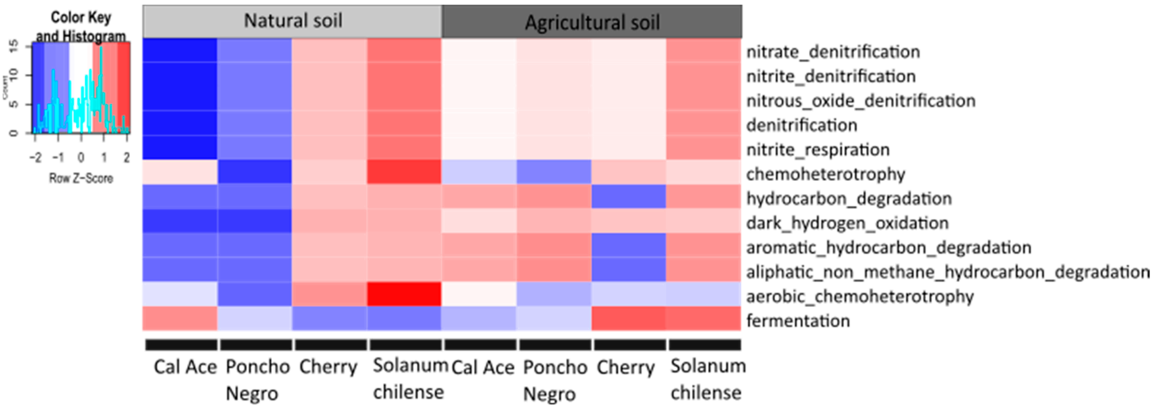

Supplementary Figure S7. Functional prediction according to FAPROTAX analysis. Relative abundance heatmap, showing functional groups that differ significantly between samples (Fold-Change 2, *p-value* < 0.05).

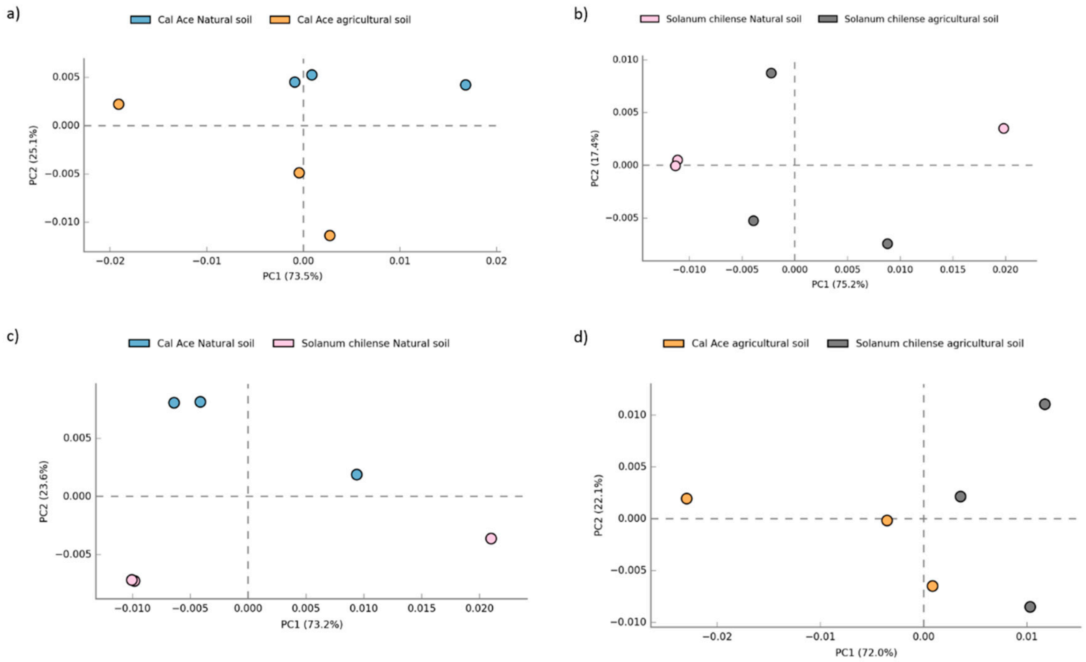

Supplementary Figure S8. Comparison of pathway prediction profiles between bacterial microbiota of selected genotypes visualized through PCA. (a) 'Cal Ace' in natural and agricultural soils; (b), *S. chilense* in natural and agricultural soils; (c) 'Cal Ace' and *S. chilense* in natural soils; (d) 'Cal Ace' and *S. chilense* in agricultural soils.

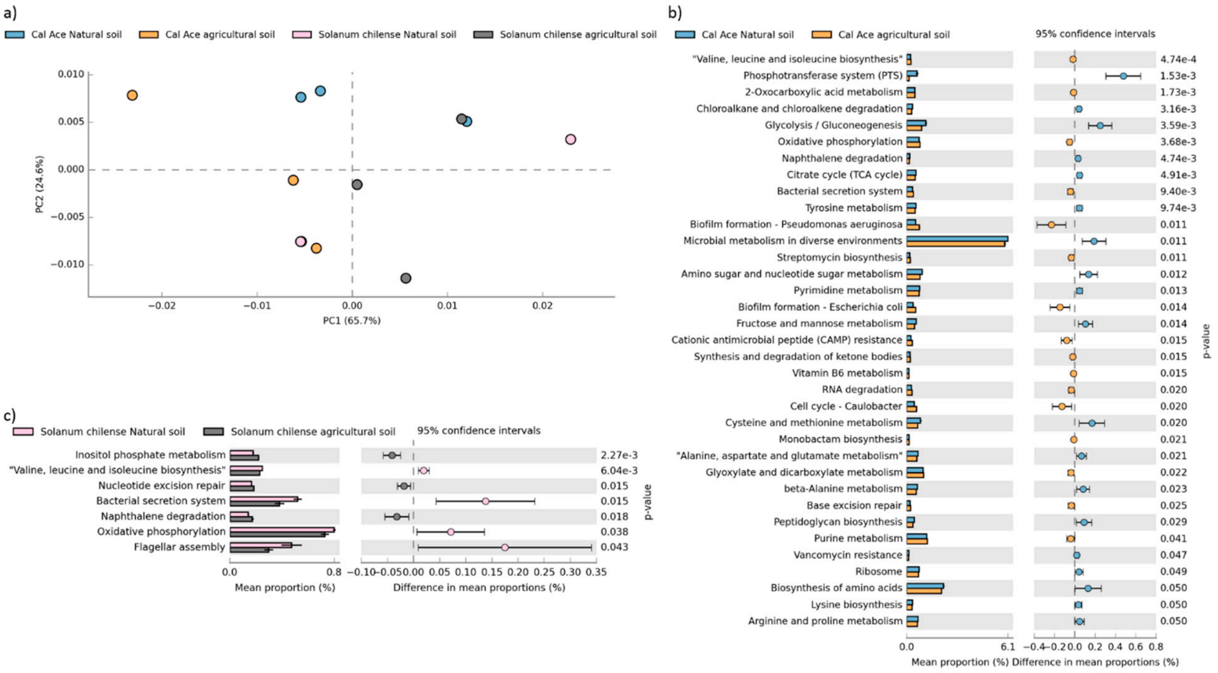

Supplementary Figure S9. Comparison of pathway prediction profiles between bacterial communities of selected genotypes. (a) PCA of pathway predictions between 'Cal Ace' and *S. chilense* in both Soil types. Mean proportion (%) and differences in the mean proportions (%) of the predicted pathway of bacterial communities of the (b)'Cal Ace' and (c) *S. chilense* genotypes in natural and agricultural soils.
